# What is Trained Develops! Theoretical Perspective on Skill Learning

**DOI:** 10.3390/sports5020038

**Published:** 2017-06-15

**Authors:** Hermundur Sigmundsson, Leif Trana, Remco Polman, Monika Haga

**Affiliations:** 1 Department of Psychology, Norwegian University of Science and Technology, 7491 Trondheim, Norway; Hermundur.sigmundsson@ntnu.no (H.S.); leiftrana@hotmail.com (L.T.); 2 Department of Sport Science, Reykjavik University, 101 Reykjavik, Iceland; 3 School of Exercise & Nutrition Sciences, Queensland University of Technology, Brisbane, QLD 4059, Australia; remco.polman@qut.edu.au; 4 Department of Neuromedicine and Movement Science, Norwegian University of Science and Technology, 7491 Trondheim, Norway

**Keywords:** children, development, probabilistic epigenesis, Neural Darwinism, dynamic systems theory, task specificity, learning

## Abstract

Knowledge about developmental theories is important for experts or specialists working with children following normal development and children who have various kinds of dysfunction, in order to better understand what happens with processes associated with motor behavior. In this article, we have explored how theories of development and learning can be used to understand processes associated with motor behavior. A probabilistic perspective emphasizes that the changes taking place in the development is a result of interaction: structural changes in the nervous system leading to changes in function and behavior and opposite, functional changes resulting in changes in structure. This bidirectional interaction between biological and experiential aspects is a continuous process which cannot be reduced to either organism or environment. Dynamical systems theory (DST) emphasizes that it is the interaction between the person, the environment, and the task that changes how our movements are, also in terms of how we develop and learn new movements. The interplay between these factors will, over time, lead to changes in motor development. The importance of experience is central to Edelman's theory of neuronal group selection (NGST). Activation of the nervous system increases the connections between certain areas of the brain, and the selection processes in the brain are a result of enhancement of neural connections involved in a "successful" motion. The central nervous system adapts its structure and function in response to internal and external influences, and hence neural plasticity is a prerequisite for learning and development. We argue that Edelman´s approach supports the theory of specificity of learning. From the perspectives of probabilistic epigenesis, DST, and NGST, we can see that being physically active and having the opportunity to get different movement experiences are of great significance for promoting motor development and learning. A variation of purposeful or rewarding physical activity in a variety of contexts will provide individual opportunities for changes of behavior in terms of both quantitative and qualitative changes in motor development.

## 1. Introduction

Research on how we acquire new motor skills has been of major significance for our knowledge of human development [1,2]. Assessing our ability to perform movements is useful because it provides a picture of how the nervous system works and whether it works in an optimal way [3]. The importance of assessing motor development as part of overall neuropsychological and developmental assessments has been a relatively common practice [4,5]. Moreover, motor development has gained increased attention because of its association with other aspects such as the function and development of cognitive, social, and emotional domains [6,7].

Although motor development is central to the overall development of children and adolescents, it was not until the beginning of the last century that scientists started to examine this relationship. To that end, there have been few theories on motor development, and progress in the field has been relatively slow. However, theories on motor development have changed and have adopted changes in thinking in disciplines such as psychology, embryology (fetal development), and biology. 

One of the more prominent general theories on development, based on probabilistic epigenesis, has been put forward by Gottlieb [8]. This ecological theory suggests that the complexity of development can be best understood as an interaction between the (biological) individual and environmental factors. Edelman’s [9,10] theory of ‘neuronal group selection’, on the other hand, tries to explain development and learning through the principles of neural brain development. Both of these theoretical approaches to motor development and learning will be discussed in more detail in this paper.

A theory which had a profound influence in the field of motor development is dynamical systems theory (DST). The primary focus of this theory is the dynamic interaction between the (moving) individual, the movement task to be carried out, and the environment this takes place in [11,12]. Any movement and development of movements according to DST not only depends on the body to perform the movement, but also the body's interaction with the environment and the interaction between these inner and outer frameworks. In this way, DST is a useful perspective to describe, explain, and predict motor development and control [13,14]. In this context, this paper will also further explore Newell's model of factors that influence the development of coordination and control of movements.

Before outlining in more detail the theories on development and learning, we have to describe and define some of the terminology. Motor development can be defined as “the process by which an individual progresses from simple movements to complex motor skills” [13] (p. 5). In line with ecological theories, the focus would be on how various factors and constraints in individual, environment, and task affects the development [13]. Motor learning, on the other hand, refers to the relatively permanent changes in movement related to experience and learning [15]). These two concepts are similar, but one can say that motor development is more related to age compared to motor learning, as motor development includes growth, maturation, experience, and learning [12]).

Henderson and Sugden [16] have proposed the concept of motor competence. Motor competence can been understood as a person's ability to perform various motor actions. The term includes both fine motor skills/activities, including coordination of small muscle movements, such as when we move fingers, and gross motor skills/activities that involve the coordination of large muscle groups and whole body movements. In order to have good motor competence, an individual must be able to master many different kinds of motor skills. How we move and what qualities our movements must have depend on the situation we are in and the movement task to be solved. Sometimes we perform precise, repetitive, and rapid movements while other times the movement task places greater challenges on the balance and control of the body to meet the stability demand of the task. In tests measuring motor development and motor performance, it is a common to assess the skills in the following categories: manual skills, eye-hand/foot coordination, object control skill, locomotor skills, and static/dynamic balance [17].

## 2. Development 

### Gottlieb

Gottlieb [18] argues that there is a continuous interaction between our genes and the environment throughout our life that guides development of individuals. We adjust to the new challenges that arise in a given environment. These adjustments are called adaptations in the theory of evolution and Gottlieb argues that these are the result of the synthesis between nature and nurture [19]. These changes come from ambient influences on the individual during its development from embryo to adult. ‘Normal’ development, according to Gottlieb, depends on stimulation. However, this stimulation varies for each individual and depends on the experiences we gain through our upbringing and development.

Gottlieb argued that for the nervous system to develop in a normal way it needs experiences of both internal and external stimulation. This stimulation must come both from the activity between neurons in the brain, and from outside ourselves such as the environment [8,20]. Our behavior and stimulation from the environment, in turn, affects which of our genes are expressed. Gottlieb called this interaction between behavior, environment, and genes ‘probabilistic epigenesis’ [8,19,21,22]. Epigenesis refers to the notion that certain developmental stages must be activated and executed before the next step can begin [18]. The process is probabilistic because one does not follow a specified format. At any stage, environmental influences can cause changes [8,18]. This is not dissimilar to ideas by Darwin [23], where development is seen as having many different influences that will also have their part in determining how the individual is formed. The idea behind this model is that DNA produces proteins, which form the building blocks of organic matter [18]. These molecules will, in turn, be influenced by environmental factors that make them either inhibit or promote the formation of other types of proteins [24].

**Figure 1:**
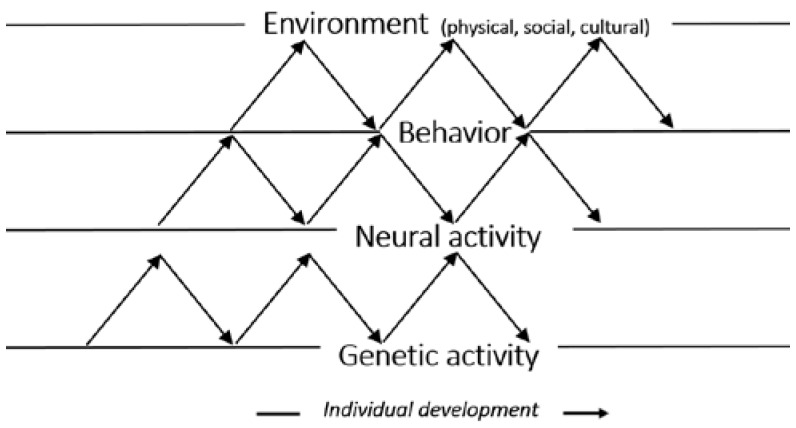
Probabilistic epigenesist. The figure illustrates the bidirectional interaction between genetic activity, neural activity, behavior, and environment in this perspective.

Probabilistic epigenesis allows for the inclusion of an individual’s upbringing and gives this equal importance as genes when it comes to how a person develops into an adult and independent person. Figure 1 provides a model of Gottlieb's theory.

Gottlieb’s theory explains how evolutionary mechanisms work at the individual level and how the environment affects our biological template DNA. The environment selects which genes are activated through the action of proteins which either promote or inhibit an individual’s different genes [18]. Probabilistic epigenesis provides an explanation for how the brain, one of the most complex and adaptable structures we know of, integrates and learns from our experiences with our environment or our past performances.

The next theory deals with how to take this understanding into brain structure and how this adaptation actually occurs in response to various coping situations.

## 3. Learning 

### Edelman

No individuals are identical. It is a necessity through evolution and development that we may be similar but not identical [10,19,21,22,25]. Edelman examined how selection worked on the body’s cells and argued that this process occurred throughout the body, especially in the brain when it was subjected to experience and learning [10,26]. Edelman [10] developed the theory of Neural Darwinism, which suggests that the way the brain develops is similar to the selection in human evolution. For the selection mechanism to function, there must be a population to select from. It is well established that the brain has numerous neuronal groups. These areas have been mapped by Brodmann. The so-called Brodmann areas are distinguishable by different types of neurons and clear ‘boundaries’ [27]. Edelman [28] has focused on these divisions and argues that they influence brain functioning and development. Hence, humans are born with a large repertoire of neurons. This variation allows selection by weeding out inactive cells [29]. Experience and learning will fine-tune these neurons into groups that together form the basis for further learning and (skill) development [9]. Edelman based his ideas on research focusing on how the brain forms distinct groups when he formed the foundation for what he calls the theory of “neuronal group selection” (NGST) [10,26,28]. Consistent with Gottlieb’s probabilistic epigenesis [8,19,21] NGST suggests that the brain forms various networks based on an individual’s experience and development. Groups of neuronal connections form a repertoire of behavioral patterns and connect different parts of the brain together [10,27,30]. Together these neural groups create networks leading to connections and thoughts. The mind is a result of these links. The large number of synaptic connections is what gives us all that we are, from motor behavior to consciousness. Neuronal organization is the core of NGST. The theory has three core elements: (1) How brain anatomy evolves and is formed from conception; (2) How the brain network forms, depending on the stimuli and experience; (3) How these networks communicate among themselves, forming overall impression and behavioral repertoires.

Core element 1 of the theory provides information on how the brain develops and the neurons come together and connect to each other. During development, the body’s cells are formed through a process of differentiation where the cells occupy their specific roles, such as skin cells or neurons. The selection procedural mechanism suggests that through stimulation and experience, those cells which are functional will be retained and those which do not find their place will disappear through cell death. It has to be kept in mind that there are neurons with different characteristics that are designed to work in networks [30,31]. “However, the final specification of the neuron and determining how it will function depends to a high degree on genetic specific influences from other neurons in the environment and appropriate use of these neurons in the network. Therefore, the nervous system development and final performance will depend on an interaction between genetic and external factors” [27] (pp. 143–144). Environmental influences form the framework for the evolutionary theory of Edelman take into consideration how at the micro level the brain’s neurons grow or die depending on the stimulation they receive from the environment.

Core element 2 of the theory provides an explanation for how neurons form networks and groups based on their experience. We are born with many neurons and connections, and more neurons are formed in the first years of life [27]. Meanwhile, neurons that do not find their place to connect with others eventually disappear—the “use it or lose it” principle [32]. The genetic code for development does not tell us the specificities of brain networks, but will provide specific restrictions on its formation [10,26,28]. “Even with such constraints, genetically identical individuals are unlikely to have identical wiring, for selection is epigenetic” [10]. Epigenetic means that an individual does not develop from the genes alone, but in each stage of development other factors also have developmental effects [21]. These mechanisms will in the end form a repertoire of groups which form the neural basis of both skill and learning. 

Core element 3 of NGST is “reentrant connections”. “Reentry” is the continuous signaling from one brain region to another and back again through massive parallel fibers of which there are many in the brain [26]. The neuronal groups of the brain interact with each other in the perception of stimuli. These neuronal groups form larger areas in the brain called cortical maps. A cortical map is specific to a type of signals, i.e., they specialize for a specific input [27]. When several different maps in the brain are topographically connected, there is no need for any overriding function or central mechanism that interacts and organizes impressions [10,28]. Our perception of the world becomes coherent and consistent through a summation of all the activity in the different areas.

Since these areas interact through what Edelman calls reentry, they will organize themselves through the strengthening and weakening of neural pathways, along with an overall map that integrates and filters these stimulations, resulting in the perception of a coherent world [30,31]. Different areas are “talking” to each other, providing overall brain function across geographical areas of different neuronal groups. As such, one can interpret the theories by Gottlieb and Edelman as a comprehensive explanation of how the brain develops and acquires learning. Throughout development, neurons and neural paths are selected and strengthened depending on their use, such as evolutionary thinking tells us.

## 4. Theories of Motor Development and Learning

In recent years, it has been widely accepted that motor development is related to both biological and environmental conditions which interact with each other. In particular, DST has adopted this perspective and started to ask pertinent questions like ‘why’ motor development takes place, and what is it that makes the developmental process occur in the way it does [3]. Based on principles of ecological theory, DST emphasizes that motor development is an interaction between several factors in the individual, the environment, and the movement task being performed [3,33,34]. The process of motor development is seen as probabilistic [35,36], but there are different factors in the environment or in the individual who together affect the probabilities that the development takes a certain direction [12]. This way of thinking is closely aligned to Gottlieb’s probabilistic epigenesis, as it emphasizes the interaction between genetic activity, neural activity, behavior, and environment.

**Figure 2:**
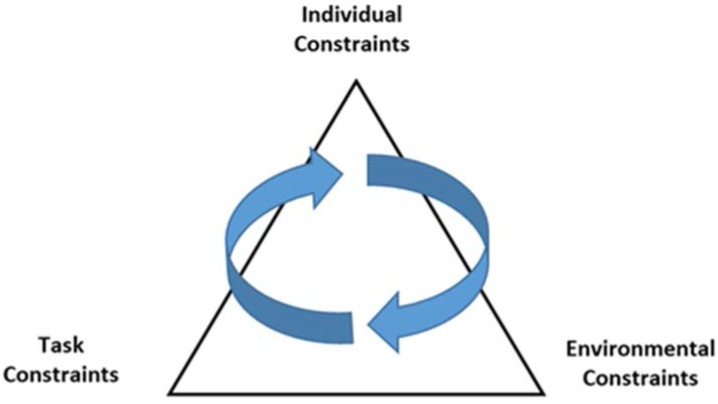
A figure of Newell's model of factors that influence the development of coordination and control of movements.

Newell´s [11] model (see Figure 2) shows how the different factors affect an individual’s movements in a reciprocal way. This model can also be used to understand both the development and learning of movements. At the top of the triangle we find individual constraints, which are conditions that are found within the subject (such as the person's height, weight, experience, and self-perceptions). Environmental constraints are found outside the body, and they can be natural (e.g., temperature, gravity, surface) and socio-cultural (e.g., the family structure, social values). Similarly, there exist certain constraints in the task, such that the movement or activity has goals or rules that must be followed. What skills and how fast the motor development/learning occurs, such as when the child learns to throw a ball, and how good they are in this skill, depends on conditions both in the environment and in the individual (such as stimuli, muscle power, and motivation). Thus, action is generated through interaction between various constraints that may be present in the individual, the surroundings, or in the individual movement task. Newell [11] emphasizes that it is the interaction between the person, the environment, and the task which changes movement, and how this interaction takes place, over time, will lead to changes in motor development/learning.

Constraints or conditions can be defined as "all conditions that are helping to reduce the number of degrees of freedom in a movement" [37]. Various factors may therefore affect how we reduce the complexity of a movement. This allows the body to be a controllable system so that we can regulate and coordinate our movements. These framework conditions may in some cases limit and reduce certain movements and movement behavior, and promote and facilitate the movement of others. The different operating conditions change the number of degrees of freedom in which the movement task can be executed. According to DST, this is a self-organizing process. A good example to illustrate Newell’s model is the development of the overarm throw. Many children master this skill before the age of 12. However, there are large individual variations in the mastery of this skill. This can be expressed in terms of having difficulty with the technique (high arm) or in terms of the outcome (direction/speed). Individual constraints like gender, age, and biological factors including muscle strength and arm mass all influence overarm throwing in children [38]. Task constraints like the requirement to throw fast or with accuracy over a long or short distance and instruction also influence movement execution. Finally, environmental constraints like the size of the object/ball to be thrown (different ball size in youth sport, for example) or the surface we are standing on has an influence on how we throw. By manipulating constraints, we can thus alter how the movement is organized and carried out.

A concept which was introduced earlier, degrees of freedom (DOF), is also important here and requires further elaboration. Our body has countless ways in which it can execute movements. It is therefore important in the learning process to have the body forced or directed to act together in so-called coordinative structures or synergies [39]. To Bernstein, the learning of new motor skills is the process of resolving the problem of coordinating all degrees of freedom in the body. He proposed three different stages in the learning process, where the organizations in synergies were resolved in different ways at each stage. The first stage he called freezing the DOF. At this stage, the number of DOF is reduced so that it becomes easier to control the remaining DOF. Referring back to the overarm throw, we see that this fits well with what characterizes the performance of a novice in practice [3]. The arm does not swing backwards, and the ball is thrown by a relatively straight arm. There is also little rotation of the upper body and the legs are held generally stationary. One can say that the throwing movement is simple in that it is mainly the arm which is moved, in this way the movement is easier to control but also less flexible and complex. The next stage is known as releasing the DOF. Now several DOF are released, making the movement more difficult to control but more flexible and effective. As the individual becomes more experienced and skilled at controlling the relatively simple movement in the first stage, the individual can release more degrees of freedom. It is possible to see that the arm will swing more backwards, the pelvic and upper body rotate as a unit, and the opposite leg of the throwing arm takes a step forward. This is a more advanced way to throw because you have to control both legs, torso, and the arm relative to each other. In the final step, the individual utilizes biomechanical aspects, such as internal or external forces which make the movement more economical and efficient. When the individual is at this stage, the arm is passed down and back across the waist, the rotation of the pelvis and torso are differentiated, and the force from the leg can be exploited in order to get the most force in the throw as possible. One can lead the arm far behind or utilize a counter movement, where the arm is passed rapidly back before the throw (taking advantage of the positive aspects of a central movement) [13].

## 5. The learning Process

Edelman’s theory on experience based selection attempts to describe the changes and adaptations that occur in the nervous system based on development and experience. The theory argues that the experience and stimuli create increased connections in specific brain areas. Training strengthens the neural connections that are used. Every time we perform a motor task it will strengthen the nerve pathways that participated in the exercise relative to the nerve connections that were not included, but only if this result is interpreted as positive [10]. It could be argued that Edelman’s theory supports the perspective of specific training; each skill is specific and should be specifically trained [40,41,42].

**Figure 3:**
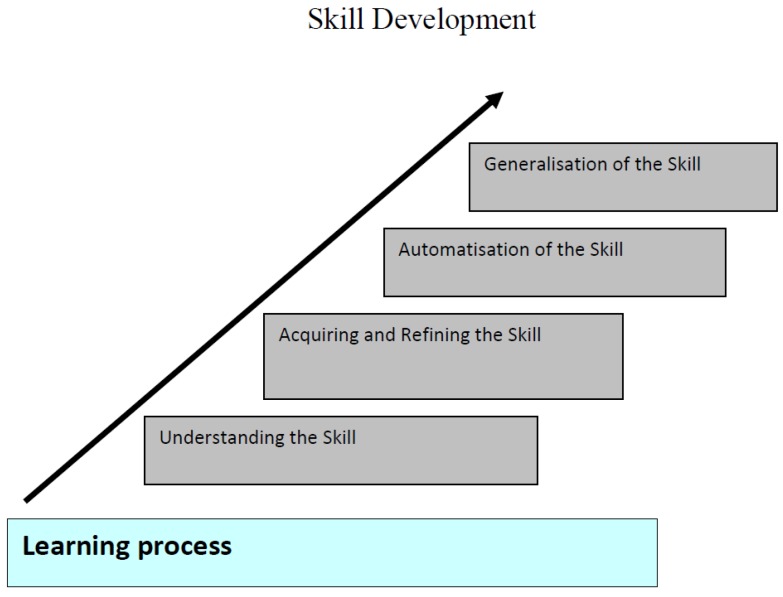
The figure above presents the learning process which is comprised of four phases. The phase we are at depends on how much we have been practicing and how much experience we have.

Figure 3 depicts the learning process which comprises four phases. It builds on Henderson and Sugden’s [16] approach of the process that occurs when learning. It is important to remember that such a division into various phases makes it easier to illustrate what happens in the learning process. In fact, the phases are more likely to overlap without distinct boundaries. Where we are in the learning process depends on how much training and experience we have in relation to what we should learn. In line with Gottlieb and Edelman theories, the experience we get through the actions and behaviors will shape the nervous system’s function and structure throughout life [43]. In the context of skill development, both quantitative and qualitative changes occur. Quantitative changes involve the development of new skills (the focus is not on the quality of the skill). This relates to the understanding of the skill (see Figure 3). The individual attempt to understand what the task requires, its purpose, how it should be carried out, and what strategies to use. Learning is experimental and the individual needs to discover, or to be explained, “rules” for task execution [44]. The individual may possess many skills that are at this level. One could say that the individual has established various networks in the brain linked to the various skills, but this network is not stable yet.

In the ‘acquiring and refining’ stage, qualitative changes in skill execution take place (i.e., one is able to control the skill to make it more efficient, reducing inaccuracies and large variations in performance). The individual will have a clearer idea of how the task can be solved, but both performance and outcome of the movement is qualitatively not stable yet. Practically speaking, the trial and error process requires much rehearsal. In the brain, the neural networks will be strengthened through trial and errors of specific tasks which will gradually lead to the learning of an effective movement solution [12,33,45]. Feedback is central to this phase. Giving frequent positive feedback when the individual does something good works well, and mastery of the tasks that an individual has been given will be enhanced through motivation, self-esteem, and coping skills, thus encouraging them further in the learning process [46]. In addition, self-monitoring can help the progress of acquisition during this stage. Both positive feedback and self-monitoring seems to be the key principles of the learning of new skills [46]. 

Following significant practice, an individual will reach the ‘Automation’ stage. At this point, the skill is highly learned, requiring little attention for its actual execution. Central to achieving this level of motor skill proficiency is the intensity or the number of repetitions of practice. Stimuli must be repeated often enough for it to lead to an organization of neural networks [10,42] and the automatization of the skill. In this context, it is therefore important for teachers/trainers to decide what skills you want the individual to master and build in sufficient practice.

In the final phase, skills may be applied or transferred to other situations or a new context. However, this will only happen if the skill has been well learned and maintained [44]. This is consistent with the brain's ability for plasticity (formability). If you do not maintain specific brain functions, these areas could degrade [42]. Previously learned skills can actually be forgotten. If you do not maintain your various skills over time, future performance levels will be inferior. For example, if a long time has passed since you have gone skiing, it takes a while before you feel that the balance and technique is in place again. The individual must establish an understanding that the skill can be used in other situations and contexts. Some individuals may have difficulties in achieving the ‘Generalization’ stage [16,17], possibly because they have not automated the skill sufficiently due to individual constraints or lack of practice [47].

Throughout the learning process, individuals will be focused on various aspects of the movement/skill and will develop an ability to self-evaluate the consequences of their performance. For progression to take place, it is important that action challenges are matched to action capabilities (skills). Determination of an individual’s stage of skill development can help coaches or teachers to provide optimal challenges. Introduction by progressively focusing on more difficult tasks (action challenges) will ensure that individuals can master the task [48,49].

Significant empirical evidence now exists supporting the notion that learning is specific. For example, the correlation between different motor tasks are generally low [50,51,52,53,54]. Drowatzky and Zuccato [52] provided support for the specificity of learning some time ago when they found only very low correlations (0.03 to 0.31) between six different balance exercises. These findings suggest that a person can be good in one balance task but not in another balance task. More recent studies have confirmed the notion that balance is very task specific [55]. This study showed that two groups of young adults who trained various balance tasks only showed progression on the task they practiced but not others. Also, there was no transfer between the balance tasks.

These findings suggest that balance consists of a number of different skills with performance varying form one balance skill to the next. Low correlations have also been observed in manual and ball skills among four-year-old children [56]. Specificity of learning motor skills is also supported by research on motor insecure children. Interventions based on the principles of specificity show better effects on motor development in comparison to other approaches [40,57]. Low correlations between different tasks are also found within mathematics and reading. For example, (r = 0.27) among 10-year-old students [58]. With regard to reading, a correlation of 0.44 between designating small letters in 1st grade and reading in 5th grade, and low correlations (0.26) between the reading of individual words in 1st grade and writing in the 5th grade have been reported [59].

Specificity of learning seems to happen in both the cognitive and motor domain, indicating that learning is relatively independent and specific [54,58]. Edelman’s NGST also supports the specificity of learning hypothesis. Training on a specific task will strengthen the neural connections involved in that particular task and thereby increase the likelihood that this behavior is executed in the future [34].

In the 1960s, it was argued that perceptual-motor functioning could improve academic performance [60,61]. The underlying assumption being that there is a general ability that is relevant in both the motor and cognitive domain. However, in their meta-analysis, Kavale and Mattson found that cognitive skills did not improve with motor training. In addition, low correlations not only exist within a specific domain but also between different domains. However, there is a growing resurgence on the association between motor and cognitive skills. Indeed, there is more recent evidence that greater mastery of fundamental motor skills is associated with enhanced academic performance [62]. However, it is unclear whether this is a direct effect or whether children who have better fundamental motor skills are more likely to be more physically active. Hence, regular and acute bouts of exercise are associated with enhanced cognitive performance (see, for example, [63] for a review).

## 6. Summary and Conclusions

This paper has interpreted and synthesized theoretical constructs around motor learning with the neuroscience of development and learning. Marrying these two research areas is significant for the clinical application of movement science to promote optimal movement development and physical health in growing children. 

A probabilistic perspective emphasizes that the changes taking place in development are a result of interactions between the individual and their environment. Subsequent structural changes in the nervous system lead to changes in function and behavior and functional changes result in changes in structure [18]. This continuous bidirectional interaction between biological and experiential events cannot be reduced to either organism or environment [64]. Newell [11] adds that it is the interaction between the person, the environment, and the task that changes our movements, including how we develop and learn new movements. The interplay between these factors will, over time, lead to changes in motor behavior. The importance of experience is also central to Edelman's theory (NGST; [9,10]). Activation of the nervous system increases connections between certain areas of the brain, and the selection processes in the brain are a result of the enhancement of neural connections involved in a "successful" motion. The central nervous system adapts its structure and function in response to these internal and external influences, and hence neural plasticity is a prerequisite for learning and development [42]. Edelman’s approach also supports the theory of specificity of learning, i.e., what is trained develops. From the perspectives of probabilistic epigenesis, DST, and NGST, it may be highlighted that a variety of purposeful movement experiences in a variety of context are of great significance for promoting motor development and skill acquisition, as doing so will provide individuals with opportunities for quantitative and qualitative changes in motor behavior and development.
